# A C57BL/6J Fancg-KO Mouse Model Generated by CRISPR/Cas9 Partially Captures the Human Phenotype

**DOI:** 10.3390/ijms241311129

**Published:** 2023-07-05

**Authors:** Ronak Shah, Paul C. M. van den Berk, Colin E. J. Pritchard, Ji-Ying Song, Maaike Kreft, Bas Pilzecker, Heinz Jacobs

**Affiliations:** 1 Department of Tumor Biology and Immunology, The Netherlands Cancer Institute, 1066 CX Amsterdam, The Netherlands; r.shah@nki.nl (R.S.);; 2 Mouse Clinic for Cancer and Aging Transgenic Facility, The Netherlands Cancer Institute, 1066 CX Amsterdam, The Netherlands; 3 Department of Experimental Animal Pathology, The Netherlands Cancer Institute, 1066 CX Amsterdam, The Netherlands

**Keywords:** fanconi anemia (FA), FANCG, interstrand crosslink (ICL), DNA damage response, genetically engineered mouse model (GEMM), CRISPR/Cas9, cisplatin (CsPt), Mitomycin C (MMC), hematopoiesis

## Abstract

Fanconi anemia (FA) develops due to a mutation in one of the FANC genes that are involved in the repair of interstrand crosslinks (ICLs). FANCG, a member of the FA core complex, is essential for ICL repair. Previous FANCG-deficient mouse models were generated with drug-based selection cassettes in mixed mice backgrounds, leading to a disparity in the interpretation of genotype-related phenotype. We created a *Fancg*-KO (KO) mouse model using CRISPR/Cas9 to exclude these confounders. The entire *Fancg* locus was targeted and maintained on the immunological well-characterized C57BL/6J background. The intercrossing of heterozygous mice resulted in sub-Mendelian numbers of homozygous mice, suggesting the loss of FANCG can be embryonically lethal. KO mice displayed infertility and hypogonadism, but no other developmental problems. Bone marrow analysis revealed a defect in various hematopoietic stem and progenitor subsets with a bias towards myelopoiesis. Cell lines derived from *Fancg*-KO mice were hypersensitive to the crosslinking agents cisplatin and Mitomycin C, and *Fancg*-KO mouse embryonic fibroblasts (MEFs) displayed increased γ-H2AX upon cisplatin treatment. The reconstitution of these MEFs with *Fancg* cDNA corrected for the ICL hypersensitivity. This project provides a new, genetically, and immunologically well-defined *Fancg*-KO mouse model for further in vivo and in vitro studies on FANCG and ICL repair.

## 1. Introduction

Fanconi anemia (FA) is an autosomal recessive human disorder that was first described by Guido Fanconi and is characterized by aplastic anemia, skeletal defects, growth retardation and a predisposition to cancer [1,2]. FA arises due to a gene mutation in the FA pathway [3]. The pathway resolves interstrand crosslinks (ICLs). It is regulated by 23 complementation groups, of which eight proteins (FANC-A, B, C, E, F, G, L, M), in association with other FA-associated proteins (FAAPs), form a ubiquitin E3 ligase complex called the FA core complex [4,5,6]. This complex assembles at the site of the ICL and monoubiquitinates the FANCI-D2 complex [5,7]. Thereafter, a cascade of proteins is recruited to resolve the ICL in an error-free manner [8].

Human *FANCG* is localized to chromosome 9 and was identified to be identical to *XRCC9*, which complemented the MMC-sensitive Chinese hamster mutant UV40 [9,10,11]. Murine FANCG protein is 83% similar to its human counterpart and its expression in human *Fancg*-deficient lymphoblasts corrects their sensitivity to crosslinks [12]. The protein predominantly localizes to the nucleus and has two leucine zipper motifs [13]. The protein is most expressed in the testis, spleen, and thymus [12]. FANCG interacts with FANCA and FANCC [14,15], and is required for the assembly of the FA core complex [16,17] and the HR-mediated repair of specific double-strand breaks (DSBs) [18].

Genetically engineered mouse models (GEMMs) are an invaluable source to study human diseases as well as uncover the physiological relevance of genes and their regulatory regions in a systemic setting [19,20]. FA is phenotypically heterogenous, and mouse models have since been generated to recapitulate the FA patient phenotype and understand the relevance of each FA gene in maintaining tissue homeostasis [2,4,21,22,23]. These GEMMs also provide a rich source to establish primary as well as transformed cell lines enabling an in-depth in vitro characterization of FA proteins and their interactome as part of the DNA damage response (DDR) network. All FA mouse models display reduced fertility and cell line-based hypersensitivity to ICLs [4,21]. Additionally, most models also demonstrate a partial embryonic lethal phenotype, but severe anemia, bone marrow failure and risk of cancer, as observed in FA patients, are not recapitulated in mice, which likely relates to differences in metabolism, life expectancy and the use of alternative ICL repair pathways [21]. Nevertheless, these GEMMs provide a solid platform to screen and test for new therapies, including immunotherapy, that have the potential to improve current standard treatments.

The improvements in genome editing via the clustered regularly interspaced short palindromic repeats (CRISPR)/CRISPR-associated (Cas) protein 9 system over the past decade have been phenomenal [24,25]. Borrowed from the prokaryotic cells, the system consists of one or more sequence-specific guide RNAs (gRNA) that identify the target DNA sequence using protospacers adjacent motifs (PAMs) and guide the Cas9 nuclease to cleave this target sequence [26,27,28]. This results in DSBs that are subsequently repaired, introducing the required genetic modification. CRISPR-Cas9 is highly specific, easy to engineer and a multiplexable tool, thereby reducing the time and cost of making models, especially animal models [29,30].

Former *Fancg*-deficient mouse models have been generated on mixed backgrounds, using selection cassettes and a partial disruption of the *Fancg* locus [31,32], presenting genotype–phenotype disparities up for discussion. Furthermore, given that immunotherapy is at the forefront of new treatment modalities in genetically unstable cancers, the genetic background of these GEMMs becomes relevant in excluding genetic confounders. To accomplish this goal, we took advantage of the genetically and immunologically well-characterized C57BL/6J inbred strain [33]. We generated a C57BL/6J *Fancg*-KO (KO) mouse model using CRISPR/Cas9 technology that employed two RNA guides that induced targeted double-strand breaks (DSBs) at each end of the *Fancg* locus. The GEMM was generated and maintained on an immunologically well-defined C57BL/6J background to exclude any confounding phenotypes related to mixed genetic backgrounds. Successful CRISPR/Cas9 mediated ablation of the *Fancg* locus was confirmed by DNA genotyping and cDNA expression. Homozygous mutants were born sub-Mendelian, indicative of embryonic lethality, and demonstrated infertility, hematopoietic defects and skewing of hematopoiesis towards the myelopoietic compartment. *Fancg*-KO PreB cells and mouse embryonic fibroblasts (MEFs) displayed hypersensitivity to crosslinking agents cisplatin and Mitomycin C. KO MEFs also displayed levels of γ-H2AX upon cisplatin exposure. Finally, reconstitution with a flagged *Fancg* cDNA-construct fully rescued the sensitivity of KO cells to cisplatin, further validating the C57BL/6J *Fancg*-KO mouse model for further fundamental and applied studies in FA research.

## 2. Results

### 2.1. Establishing a Genetically and Immunologically Defined C57BL/6J Fancg-KO Mouse Model

**Figure 1:**
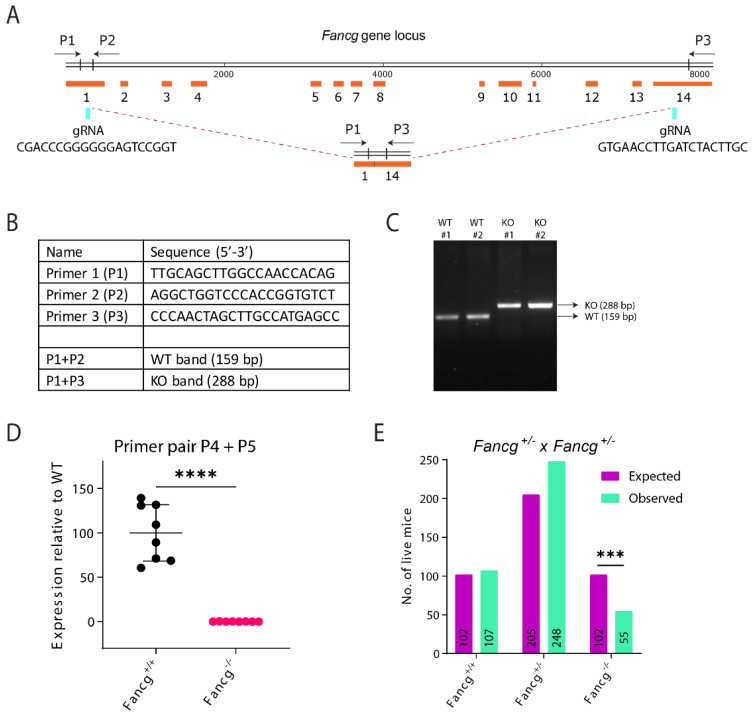
Establishing a genetically and immunologically defined C57BL/6J Fancg-KO mouse model. (**A**) Schematic representation of the *Fancg* locus in the mouse and its CRISPR/Cas9 based inactivation. Cas9 activity targeted by the two gRNAs at *Fancg* locus in exon 1 and 14 is indicated (blue flashes). The product after the 7.5kb DNA fragment deletion is also displayed and can be detected and distinguished from wild type using the three primers P1, P2 and P3 (black arrows). (**B**) The table lists the sequence of primers used to screen for the knockout of *Fancg* allele and the primer combinations used to detect WT and *Fancg*-KO band simultaneously in one PCR reaction. (**C**) Genotype PCR distinguished WT (P1+P2) from KO(P1+P3) alleles. (**D**) Using qRT-PCR the lack of exons 2, 3 and 4 in the mutant cDNA of *Fancg* was confirmed using the primer pair id 253735709c1 from PrimerBank database. GAPDH was used as internal control and expression was plotted upon normalizing to expression of GAPDH and WT allele. *p* value was calculated using Welch’s *t* test, **** *p* < 0.0001. (**E**) Observed and expected numbers of offspring obtained from *Fancg*^+/−^ intercrosses. *Fancg*-KO mice were born at sub-Mendelian frequencies. *p* values were calculated using Pearson’s Chi square test, *** *p* < 0.001.

Fanconi anemia (FA) complementation group G (*Fancg)* is a member of the FA core complex responsible for the ubiquitination of the FANCI-D2 complex. It acts as a scaffold in the formation of the FA core complex [14,16] and is the third most mutated gene in FA patients [34,35]. Murine *Fancg* is located on chromosome 4, is encoded on the reverse strand and consists of 14 exons (GeneID: 60534) [36]. The *Fancg-*KO (KO) mouse model was generated using C57BL/6J zygotes that were injected with in vitro transcribed Cas9 mRNA and two guide RNAs (gRNAs) targeting exon 1 (gRNA1; at chromosomal position −43010202 to 43010221) and 14 of *Fancg* (gRNA2; at chromosomal position 43002804 to 43002823) (Figure 1A), resulting in a complete deletion of the *Fancg* locus. This gene targeting strategy ensures a pure C57BL/6J genetic background and excludes any potential linkage-related mutations associated with long-term embryonic stem cell (ES) culturing. Following the CRISPR/Cas9 procedure, pups were genotyped by PCR using the set of three primers P1, P2 and P3 (Figure 1B), which allowed a direct distinction between wild type (WT) and knockout (KO) alleles (Figure 1C) and maintained on a pure C57BL6/J background. Further, deletion of the entire gene was confirmed by genomic PCR and the lack of expression by qRT-PCR (Figure 1D) on cDNA isolated from mouse embryonic fibroblasts (MEFs) (Supplementary Figure S1A). Intercrossing heterozygous *Fancg* mice resulted in the expected numbers of WT and heterozygous mice. On the other hand, KO mice were born at a sub-Mendelian frequency, almost 50% less than expected (Figure 1E). Interestingly, we have observed normal KO frequency at E14.5 (Shah et al., unpublished), indicating that homozygous embryos are lost beyond E14.5, perhaps due to an HSC defect that either arises around E12.5 [37] or beyond E14.5 during the transition from embryonic to adult hematopoiesis. Nevertheless, homozygous mice that survived this crisis developed normally and did not show any growth retardation.

### 2.2. C57BL/6J Fancg-KO Mice Display Stem Cell Defects

**Figure 2:**
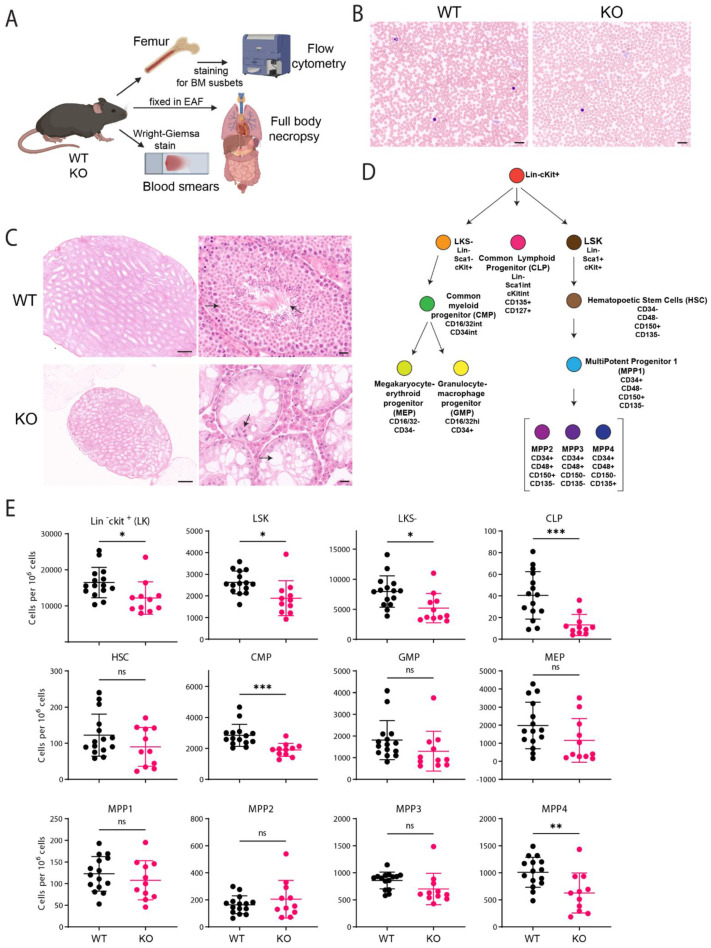
C57BL/6J Fancg-KO mice display stem cell defects. (**A**) WT and KO mice were sacrificed, and various tissues were harvested. Femur and tibia were flushed and used to isolate hematopoietic progenitors that were characterized using a special cocktail described in D using flowcytometry. Other tissues such as the reproductive organs, sternum and spleen were fixed in EAF, processed and stained for H&E. Blood smears were performed by puncturing the heart and stained with the Wright–Giemsa protocol. (**B**) Representative Wright–Giemsa-stained blood smears from WT and KO adult mice. Scale bar: 20 um. (**C**) Sections of testis from WT and KO mice. Wild type testis are much larger in size than knockout mice (left panel). On zooming in, black arrows indicate normal spermatogenesis (black arrow) in wild type mice, while knockout mice, filled only by vacuoles (black arrow) and sertoli cells, indicate severe testicular degeneration and impaired spermatogenesis. Scale bar: 100 um. (**D**) Schematic representation of the different hematopoietic subsets identified using defined cell surface markers. (**E**) Numbers of different hematopoietic subsets as defined in Figure 2A. The graphs indicate specific cell counts per 1 * 10^6^ live cells. In each graph the bar represents the mean ± sd. WT (*n* = 15) and KO (*n* = 11). *p* values were calculated using one-way ANOVA. * *p*< 0.05, ** *p* < 0.01, *** *p* < 0.001, ns indicates non-significance.

In mouse tissues, FANCG expression is highest in the testes and defects have a profound impact on the reproductive and hematological systems. To assess the impact of a *Fancg* ablation in C57BL/6J mice, we analyzed the bone marrow by multiparametric flowcytometry for hematopoietic progenitors; small intestine, thymus, reproductive organs and sternum by hematoxylin and eosin (H&E) staining; and performed blood smears on 15 WT and 11 KO mice (Figure 2A). Giemsa–Wright-stained blood smears did not reveal any differences between WT and KO conditions and displayed no signs of anemia (Figure 2B). Histopathological characterization revealed that KO mice were infertile and displayed hypogonadism, characteristic of FA mouse models [1]. For instance, KO male mice had a smaller testis as compared to WT mice (Figure 2C, left panel). Further magnification of the H&E sections revealed a complete absence of germ cells, i.e., spermatogonia and spermatocytes; only Sertoli cells could be detected in the lumen (Figure 2C, right panel). However, no major pathological alterations were observed in the sternum, thymus and spleen of knockout mice as compared to WT mice.

In FA patients, the failure to repair ICLs impairs hematopoiesis but this has not been consistently observed in FA mouse models. Reasons for these interspecies phenotypic differences relate to differences in metabolism, life expectancy and the differential activation of the alternative, although less effective, backup pathways of ICL repair [21]. To determine the impact of *Fancg* ablation on murine hematopoiesis, we applied multiparametric flow cytometry. To identify the different hematopoietic progenitors and subset populations, we adopted the gating strategy adapted from Wilson et al. [38] (Figure 2D, Supplementary Figure S1B). In normal adult mice, functional HSCs are a part of Lineage (Lin)-negative cells expressing high levels of the c-Kit receptor and stem-cell antigen-1 (Sca-1) (LSK cells) [38,39]. In comparison to the WT mice, the LSK population was significantly reduced in the KO mice (1.4-fold) mice, but the HSC population itself was not significantly reduced although a trend could be observed (Figure 2E). The common myeloid progenitors (CMPs) and common lymphoid progenitors (CLPs) also displayed a drastic reduction. Although the CMP subsets, Granulocyte-macrophage progenitor (GMP) and Megakaryocyte-erythroid progenitor (MEP), and the Multipotent progenitor (MPP) subsets, MPP1, Myeloid-primed MPP2-3 did not show any significant differences between WT and KO mice, the lymphoid-primed MPP4s [40] were significantly reduced in KO mice (Figure 2E). Thus, the lack of FANCG affects the fitness of the hematopoietic progenitors. The observed reduction in LSK cells and the shifting in the progenitor compartments from lymphoid towards myeloid lineages are indicators of stressed hematopoiesis, consistent with previous studies in other systems [23,39,41]. In summary, while histopathological analysis failed to reveal any signs of anemia, a detailed multiparametric analysis of our KO mouse model identified a bone marrow phenotype. These insights further highlight the critical relevance of the FA pathway in maintaining tissue homeostasis within the hematopoietic precursor compartment, known to be highly sensitive to endogenous, DNA crosslinking metabolites [42,43].

### 2.3. Fancg-KO Cells Are Hypersensitive to Crosslinking Agents

**Figure 3:**
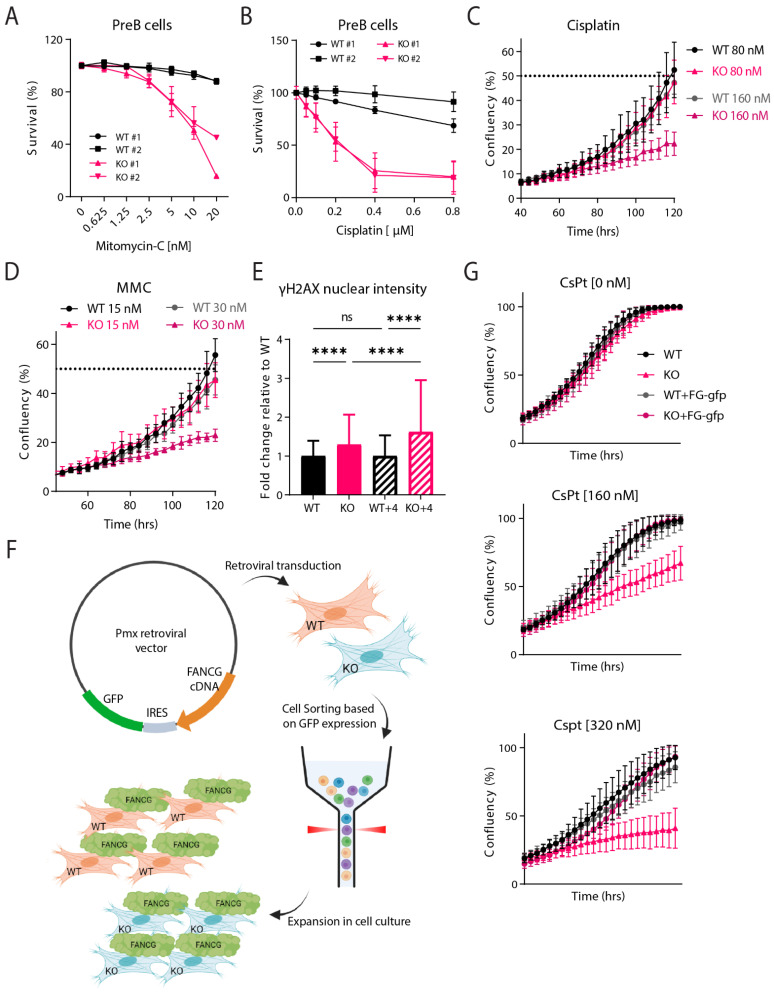
Fancg-KO cells are hypersensitive to crosslinking agents. (**A**,**B**) PreB cells cultured from independent WT or KO fetal livers were exposed to increasing concentrations of (**A**) Mitomycin C or (**B**) cisplatin and measured after three days by flowcytometry. Graph displays survival for two independent clones for each genotype, normalized to its untreated condition. Experiments were performed twice. Bar represents mean ± sd. (**C**) *Trp53*kd WT and KO MEFs were seeded at 250 cells/well in a 96-well plate and treated with two different doses of cisplatin (80 and 160 nM) a day later. Cell confluency was measured every 4 h using IncuCyte live cell imaging system from two independent experiments. Analysis was performed for the timepoint when WT samples reached 50% confluency. Dots at each timepoint indicate mean and bars represent sd. (**D**) *Trp53*kd WT and KO MEFs were seeded at 250 cells/well in a 96-well plate and treated with two different doses of MMC (15 and 30 nM) a day later. Cell confluency was measured every 4 h using IncuCyte live cell imaging system from two independent experiments. The analysis was performed for timepoints when WT samples reached 50% confluency. Dots at each timepoint indicate mean and bars represent sd. (**E**) *Trp53*kd WT and KO MEFs were seeded on coverslips and treated with 20 μM cisplatin. After 1 h, one batch of cells was fixed while the other batch was refreshed with medium and fixed 4 h later. Cells were stained for γ-H2AX and nuclear intensity was measured using a microscope. Graphs display the intensity as fold change relative to WT. *p* values were calculated using a Brown–Forsythe and Welch ANOVA test. **** *p* < 0.0001, ns indicates non-significance (**F**) Schematic displaying the protocol utilized to obtain stable WT/KO MEFs expressing the Fancg-flag-gfp overexpression construct. *Trp53*kd MEFs were retrovirally transfected with the pmx vector, sorted for GFP expression and expanded for further use. (**G**) TP53kd WT, KO, WT+FG-gfp and KO+FG-gfp MEFs were seeded at 250 cells/well in a 96-well plate. Cell confluency was measured with/without cisplatin (CsPt) every 4 h using IncuCyte live cell imaging system from two independent experiments. Dots at each timepoint indicate mean and bars represent sd.

Cells with inactivating mutations in FA proteins are characterized by their hypersensitivity to DNA crosslinking agents [6,44]. PreB cells established from independent WT and KO fetal livers were either mock-treated or treated with increasing doses of MMC or CsPt and measured using flowcytometry (Figure 3A,B). Survival analysis revealed that *Fancg-*KO PreB cells were hypersensitive to both crosslinking agents. Similarly, upon measuring proliferation dynamics using the IncuCyte system, Trp53kd *Fancg-*KO MEFs also displayed hypersensitivity to both crosslinking agents in a dose-dependent manner (Figure 3C,D).

DNA damage, including ICLs, causes replication stress that is accompanied by the phosphorylation of the histone variant H2AX at Ser 139 (γ-H2AX) [45]. Because the formation of γ-H2AX is abundant and fast, it is widely applied as a marker for replication stress and the activation of a DNA damage response (DDR) in general. To identify if the absence of FANCG and the concomitant failure to restore ICLs leads to an increased DDR, we exposed WT and KO MEFs with 20 µM CsPt for an hour and either fixed cells immediately after or after release into a normal medium for 4 h and measured γ-H2AX intensity. Already 1 h after CsPt treatment, KO MEFs displayed a significant increase in the formation of nuclear γ-H2AX as compared to WT cells. The formation of γ-H2AX further increased after 4 h indicating that defective ICL repair in KO cells resulted in the continued presence of the lesions and thereby, the accumulation of γ-H2AX foci.

To validate this model further and provide final proof of the successful inactivation of FANCG in the mouse germline, a functional Flag-tagged *Fancg* cDNA was constructed. The cDNA construct was inserted into a pmx-ires-gfp retroviral vector (Supplementary Figure S1C) allowing the co-expression of GFP and FANCG under the same promoter. Next, the vector was introduced into the WT and KO cells and GFP was used to sort FANCG-FLAG-GFP (FG-gfp) positive cells, which were expanded in culture (Figure 3F, Supplementary Figure S1D). IncuCyte experiments were repeated with the newly reconstituted cell lines (WT+FG-gfp and KO+FG-gfp) as well as the WT and KO cells. All four cell lines proliferated at the same rate, indicating that the FANCG overexpression did not hamper cellular growth. Upon increasing doses of cisplatin, KO cells, as expected, displayed sensitivity and struggled to proliferate (Figure 3G). On the other hand, the reconstituted KO cells (KO+FG-gfp) displayed normal, i.e., wild type sensitivity to this replication stressor. These data demonstrated that the hypersensitivity of the KO to ICLs could be rescued by reintroducing FANCG back into the cells. Taken together, the sensitivity to ICLs, increased DNA damage response and rescue experiments validate our C57BL/6J *Fancg-*KO model, which displays all tested characteristics of an FA defect.

## 3. Discussion

Crosslinking agents are widely applied in cancer therapy as they introduce one of the most toxic DNA lesions, i.e., interstrand crosslinks (ICLs). These lesions are effectively repaired by the Fanconi anemia (FA) repair pathway. Inactivation of one of the FA pathway components can lead to pleiotropic defects such as aplastic anemia, congenital abnormalities and skeletal retardation in FA patients. While genetically engineered mouse models (GEMMs) fail to recap all the phenotypes seen in FA patients, the common hypersensitivity to ICLs studies closely recapitulates those of FA patients and cells. In this regard, FA GEMMs and the cell lines thereof provide ideal model systems to study the relevance of FA-ICL repair in stem cell maintenance and hematopoiesis. Applying the CRISPR/Cas9 technology in C57BL/6J zygotes, we generated and characterized a novel, genetically and immunologically well-defined C57BL/6J *Fancg*-KO mouse model. To exclude the possibility of generating hypomorphic gene products, the entire *Fancg* locus was deleted. The ablation of the *Fancg* locus was verified by genotyping and RNA expression. In contrast to conventional KO approaches, this gene targeting strategy excludes experimental confounders related to mixed backgrounds and the co-segregating of modulators and other unrelated genetically linked phenotypes [31,32]. The exclusion of such confounders warrants conclusive genotype–phenotype interactions.

To minimize the genetic drifting, *Fancg* mutant mice were maintained on a C57BL/6J background. To derive homozygous mutants, heterozygous *Fancg* mice were intercrossed. KO mice were obtained at a sub-Mendelian frequency, suggesting that homozygous condition causes embryonic lethality, like recent observations made for a *Fanci^−/−^* model [22] and *Fancg^−/−^* model [37] (Figure 1E), but contrasting previous observations made in independent *Fancg* mouse models [31,32]. In these reports, *Fancg^−/−^* mice were born at a normal Mendelian frequency. However, these studies used mice with mixed backgrounds [31,32], a potential confounder that could mask specific genotype–phenotype interactions [2]. As we observe expected *Fancg*-KO numbers at E14.5 but not at weaning (E21), Domenech et al. argue that although the compensatory mechanisms reduce the differences between FANCG-deficient and WT embryos by E14.5, early HSC defects in Fancg^−/−^ embryos (E11.5-E12.5) ultimately result in perinatal lethality [37]. Nevertheless, consistent with other FA mouse models in previous studies, our KO mice were also infertile with gross germ cell defects and reduced size of testes/ovaries, which might be due to the RAC1-based exhaustion of primordial germ cells in the absence of FANCG [46]. Otherwise, *Fancg*-KO developed normally without any overt macroscopic abnormalities. Consistently, none of the tissues examined microscopically displayed any obvious pathological features.

Regarding the involvement of the FA pathway in hematopoiesis, the *Fancg^−/−^* mouse models studied thus far did not reveal hematological defects based on blood cell counts, hemoglobin analysis and blood smears [31,32,39,47,48]. Like other FA mice [23,41,49,50], the blood smears of our KO mice showed no signs of anemia indicating that unlike FA patients, spontaneous anemia inflicted by reactive crosslinking metabolites does not occur in mice [43]. Therefore, the anemic phenotype in humans might be visualized better by the disruption of more than one gene in the FA pathway in mice [39,51], as shown previously in compound GEMMs lacking *Fancc* and *Fancg* [39].

Regarding hematopoietic subsets, the bone marrow of our KO mice shows a significant reduction in early progenitors, i.e., an LSK subset, which is in line with observations made by Barroca et al. in their *Fancg*^−/−^ mice [47] and other FA mouse models [51,52,53]. We also noted similarities regarding the common lymphoid and myeloid progenitors (CLP and CMP), although we did not observe a significant reduction in the GMP and MEP subsets. While Barroca et al. use CD34 and CD135 (*Flk2*) to define their short-term and long-term HSCs [47], we utilized a more comprehensive panel including SLAM receptors [54] CD150 and CD48 to define our HSC and MPP populations as described by Wilson et al. [38]. Interestingly, their short-term HSCs (LSKCD34^+^Flk2^−^) are equivalent to our MPP1-3 and their MPPs (LSKCD34^+^FLk2^+^) are equivalent to our MPP4 populations and display similar behaviour. However, unlike their long-term HSCs (LSKCD34^−^Flk2^−^), we do not see a significant drop in our HSCs (LSKCD34^−^CD48^−^CD150^+^CD135^−^). In summary, both models revealed an important role of FANCG in myelopoiesis and lymphopoiesis. Additionally, our data demonstrate that lymphoid progenitors (CLPs and MPP4s) are compromised, likely to sustain the differentiation of myeloid progenitors GMPs and MEPs, a hallmark of stressed hematopoiesis.

The most stable FA phenotype relates to the hypersensitivity to crosslinking agents [22]. PreBs and MEFs derived from our KO mice were, as expected, hypersensitive to both cisplatin and MMC. FA patients present an increased p53-dependent apoptosis [2]. Our in vitro study was limited for p53 analysis due to immortalization of our MEFs using a p53-directed shRNA. Nevertheless, we were able to rescue the ICL sensitivity by retroviral transduction of a wild type murine *Fancg* cDNA. In addition, this construct possesses Flag tags flanking FANCG, which can be utilized to perform immunoprecipitation-based studies and provides a solution for the lack of efficient anti-mFANCG antibodies in the market for biochemistry and molecular biology-based experiments.

In summary, we present here a new, genetically, and immunologically well-defined C57Bl/6J *Fancg*-KO mouse model carrying a complete ablation of the *Fancg* locus that presents some characteristics of FA phenotypes. In addition to the mouse model, cells derived thereof as well as their reconstituted counterparts provide an opportunity to study the FA pathway, particularly the role of FANCG under specific ICL stress conditions caused by metabolites as well as chemotherapeutics.

## 4. Materials and Methods

### 4.1. Generation of Fancg^−/−^ Mouse

A *Fancg-*KO mouse model was generated wherein C57BL/6J zygotes were injected with in vitro transcribed Cas9 mRNA and two guide RNAs (gRNAs) targeting *Fancg* [30,55]. The two gRNAs targeting exons 1 and 14 were designed using the crispr.mit.edu tool and transcribed in vitro from PCR templates that had a T7 promoter. To distinguish wild type and homozygous mutants, we established a three-primer PCR strategy. Pups born were selected for the knock-out allele, backcrossed once onto C57BL6/J and maintained for the desired experimental genotype.

### 4.2. Generation of Primary Cell Lines and Cell Culture

Timed-matings of *Fancg*^+/−^ females were set up with corresponding males to obtain embryos. On day 14.5 of gestation, embryos were isolated to prepare the primary mouse embryonic fibroblasts (pMEFs). From fetal liver, PreB cell cultures were established by culturing on irradiated ST2 feeder cells in complete IMDM medium (Iscoves, supplemented with 8% fetal calf serum (FCS), 50 μM 2-mercapthoethanol, penicillin/streptomycin) supplemented with IL-7 containing supernatant [56]. Primary MEFs (2, from independent embryos per genotype) were isolated using Trypsin and cell strainers and cultured under low (3%) oxygen conditions, with 5% CO_2_ at 37 °C. To immortalize MEFs, pMEFs were transduced with a lentivirus encoding a p53-specific shRNA [57]. The immortalized *Trp53*kd MEFs were grown in complete IMDM medium under normal oxygen levels with 5% CO_2_ at 37 °C.

### 4.3. PCR Genotyping

Genomic DNA was extracted from mouse toe tips and used as template for polymerase chain reaction (PCR) genotyping. The PCR protocol was: 95 °C for 2 min and then 40 cycles of 95 °C for 30 s, 58 °C for 40 s and 72 °C for 1 min; last 5 min at 72 °C.

### 4.4. qRT-PCR

RNeasy mini (Qiagen) was used to isolate total RNA from wild type and *Fancg*-KO MEFs for qRT-PCR. The cDNA libraries were synthesized using Invitrogen Superscript III kit and random hexamer primers. *Fancg* was amplified with high fidelity PfuUltra Hotstart DNA polymerase (Stratagene, San Diego, CA, USA) using gene specific primers (id: 253735709c1) from the PrimerBank database [58].

### 4.5. Immunofluorescence of Bone Marrow (BM) Cells

Mice were euthanized at the indicated age (8–16 weeks) and BM cells from the femur and tibia were flushed out using 21-gauge syringes in cold PBEA buffer (1× PBS, 0.5% BSA, 2 mM EDTA, 0.02% Sodium Azide). Erythrocytes were eliminated from the BM cells using an erylysis buffer (NH_4_Cl, KHCO_3_, 0.5M EDTA) and passed through a 70 µm filter. Next, 10 million cells were used for staining. The following antibodies were used: Mouse Lineage Cell Detection Cocktail biotin antibody (1:40) followed by c-kit-APC (Clone 2B8, eBioscience, Sanata Clara, CA, USA), Streptavidin-APC-Cy7 (Southern Biotech, Homewood, AL, USA), CD135-PE (Clone A2F10), CD48-PE-Dazzle (Clone HM48-1), Sca1-PE-Cy7 (Clone D7), CD34-FITC (Clone RAM34, 1:100, Invitrogen, Waltham, MA, USA), CD127-BV421 (Clone A7R34), CD150-BV650 (Clone TC15-12F12.2) and CD16/32-BV786 (clone 2.4G2, BD Biosciences, Franklin Lakes, NJ, USA). Dead cells were excluded by 7AAD staining. All the antibodies for FACS analysis were from Biolegend, San Diego, CA, USA and used at 1:200, unless otherwise specified.

### 4.6. Histopathology

The spleen, thymus, sternum and testis/ovary were collected and fixed in ethanol-(not glacial) acetic acid-formalin (EAF). Samples were embedded in paraffin, sections of 4 µm thickness were made and stained for hematoxylin and eosin (H&E). The sections were reviewed with a Zeiss AxiosKOp2 Plus microscope (Carl Zeiss Microscopy, Jena, Germany), and images were captured with a Zeiss AxioCam HRc digital camera and processed with AxioVision 4.8.2 software (both from Carl Zeiss Vision, München, Germany). The scale bars were set at 50 μm. Blood smears were stained with Wright–Giemsa stain.

### 4.7. PreB Survival Assay

Survival assay was performed as described previously [56]. Briefly, 10^5^ PreB cells were seeded in 12-well plates containing an ST2 feeder layer and 1 mL complete medium and IL-7 in the continuous presence of different doses of the CsPt or MMC. To determine cell survival, cells were harvested after 3 days of culture and stained with DAPI. The number of DAPI-negative cells was measured on a Fortessa (Becton Dickinson, Franklin Lakes, NJ, USA). Data analysis was performed with FlowJo software V10.8.

### 4.8. IncuCyte Proliferation Assay

The IncuCyte ZOOM instrument (Essen Bioscience, Goettingen, Germany) live cell imaging system was used to monitor the cell growth of MEFs. In total, 250 cells were plated in a 96 Greiner micro clear black well plate and imaged every 4 h in the presence or absence of CsPt or MMC. The default- IncuCyte software SX5 parameters for a 96-well plate with a 10× objective was used for imaging. The IncuCyte software SX5 was used to calculate the mean confluence from four non-overlapping bright phase images of each well.

### 4.9. γ-H2AX Immunofluorescence

Immunofluorescence was performed as described previously. Cells were grown on coverslips for a day before the treatment with 20 µM CsPt for 1 h. After treatment, one batch was placed on ice and the second batch was refreshed with medium. After 4 h, both batches of cells were washed with PBS and pre-extracted with PBS/0.5%TritonX-100) on ice for 1 min. Cells were then fixed using 4% Formaldehyde for 15 min at room temperature (RT). Fixed cells were then incubated with primary Abs against γ-H2AX at RT for 90 min. Cells were washed and incubated with secondary Abs alongside DAPI for 1 h at RT. After washing, coverslips were mounted onto glass slides using Aqua Poly/Mount. Pictures were taken with a Zeiss AxioObserver Z1 inverted microscope using a 63× lens equipped with a cooled Hamamatsu ORCA AG Black and White CCD camera (Carl Zeiss Vision, München, Germany). Nuclear intensities were measured using a macro designed with ImageJ software 1.53t.

### 4.10. Fancg-Flag Reconstitution

Mouse *Fancg* cDNA sequence was obtained from Ensembl. The cDNA sequence was flanked on both ends by two glycine residues and a Flag tag. *Fancg*-Flag sequence was ordered as a geneblock from Integrated DNA Technologies (IDT). The sequence was cloned into pMX-IRES-GFP plasmid (Supplementary Figure S1). The orientation and insertion of the geneblock was confirmed using three primer sets (Table 1). To transduce the *Trp53*kd MEFs with *Fancg*-Flag cDNA, HEK293T cells were seeded in a 10 cm dish. The following day, HEK293T cells were transfected with 6 μL X-tremeGENE (Roche), 194 μL of serum free medium (SFM) and incubated for 5 min at RT. Next, 2 μg of pMX-*Fancg*-Flag-IRES-GFP and packaging vector (pCL-Eco) were added in a total of 200 μL Serum Free Medium. The ratio of X-tremeGENE to total DNA was 3:1, while that for the plasmid to pCL-Eco was 3:2. Both X-tremeGENE and plasmid mix were put together and incubated for 30 min at RT. Following incubation, 400 μL of the final mixture was added dropwise to each well already containing 1.6 mL of complete IMDM medium. These cells were cultured under standard conditions for 48 h after which the supernatant containing retroviral particles was collected. Polybrene^®^ (10 mg/mL) was added to the supernatant at a final concentration of 1 mg/mL. Next, 2 mL of virus supernatant was added to 500,000 MEFs. After 5 days, cells were harvested and sorted for positive GFP expression to be further used for culture.

### 4.11. Statistical Analysis

To assess the statistical significance of our data, the appropriate tests were performed using GraphPad Prism. Briefly, for qRT-PCR analysis, the *p* value was calculated using Welch’s *t* test. For the comparison between expected and observed numbers of offspring, p values were calculated using Pearson’s Chi-square test. For the hematopoietic subsets, p values were calculated using one-way ANOVA. For the γ-H2aX nuclear intensity, p values were calculated using Brown-Forsythe and Welch ANOVA tests.

## Figures and Tables

**Table 1:** Sequence of primers used to screen for the insertion of the customized *Fancg* geneblock in the pMX-IRES-GFP plasmid.

Name	Sequence (5′-3′)
pmxFG_Internal primer A fwd	GAGGGATGTCCTTCTGACTGC
pmxFG_Internal primer A rev	GAGAACCTTGTCTCTGAGCCACCC
pmxFG_Internal primer B fwd	CTGCCGTGTTGCCCAGTCTGGGTC
pmxFG_Internal primer B rev	GGCTTCGCGGTTAGGGGGATGGAT
pmxFG_Internal primer C fwd	TCTTCCACTGTATTTAGAAACCTG
pmxFG_Internal primer C rev	TTATACACGTGGCTTTTGGCCGCA

## Data Availability

Not applicable.

## Supplementary Materials

The following supporting information can be downloaded at: https://www.mdpi.com/article/10.3390/ijms241311129/s1.
