# Supplementary material for: A C57BL/6J Fancg-KO Mouse Model Generated by CRISPR/Cas9 Partially Captures the Human Phenotype
Source: Int J Mol Sci. 2023 Jul 5;24(13):11129. doi: 10.3390/ijms241311129 (PMC10342586; doi:10.3390/ijms241311129)
Supplement: Supplementary file 1 [file ijms-24-11129-s001.zip › ijms-2478066-supplementary.pdf]

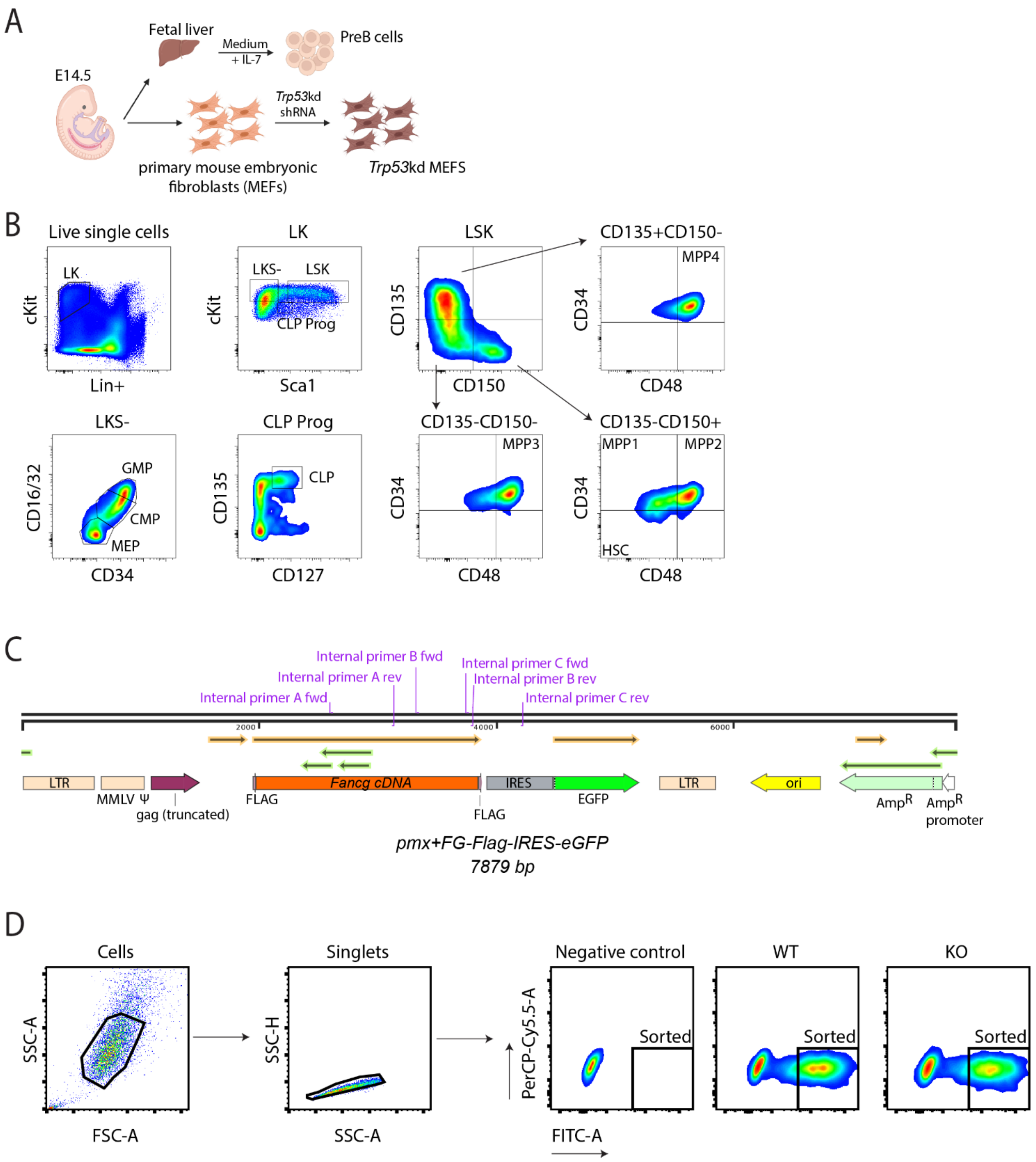

Figure S1. Characterization of the *Fancg*-KO mouse model in vivo and in vitro. (**A**) Strategy to establish PreB cells and MEFs in culture from two independent E14.5 embryos per genotype. Fetal livers were processed to grow PreB cells in the presence of an IL-7 supplemented medium and an irradiated stroma layer. Primary MEFs grown from the rest of the embryo were transduced with a *Trp53*-targeted shRNA to immortalize them in culture [57]. (**B**) Flowcytometry-based gating strategy to identify different hematopoietic subsets. Bone marrows were flushed, erylyzed (*see Materials and Methods*) and stained with a customized cocktail of surface markers and live single cells were used to identify the different subsets. (**C**) Plasmid map illustrating the *Fancg* cDNA flanked by FLAG tags cloned into a pmx-IRES-EGFP retroviral vector and the primers used for the PCRs for stable overexpression of FANCG in cells. (**D**) Flow cytometry plots for WT/KO cells transduced with the FG-gfp construct and strategy used to sort out GFP positive cells as explained in Figure 3F.
